# Source Environments of the Microbiome in Perennially Ice-Covered Lake Untersee, Antarctica

**DOI:** 10.3389/fmicb.2019.01019

**Published:** 2019-05-10

**Authors:** Klemens Weisleitner, Alexandra Perras, Christine Moissl-Eichinger, Dale T. Andersen, Birgit Sattler

**Affiliations:** ^1^Institute of Ecology, University of Innsbruck, Innsbruck, Austria; ^2^Austrian Polar Research Institute, Vienna, Austria; ^3^Center for Medical Research (ZMF), Medical University of Graz, Graz, Austria; ^4^Department of Internal Medicine, Joint Facilities, Medical University of Graz, Graz, Austria; ^5^BioTechMed-Graz, Graz, Austria; ^6^SETI Institute, Mountain View, CA, United States

**Keywords:** Lake Untersee, 16S rRNA, Antarctica, Anuchin Glacier, cryoconite holes, transfer of biota, source tracking

## Abstract

Ultra-oligotrophic Lake Untersee is among the largest and deepest surface lakes of Central Queen Maud Land in East Antarctica. It is dammed at its north end by the Anuchin Glacier and the ice-cover dynamics are controlled by sublimation — not melt — as the dominating ablation process and therefore surface melt during austral summer does not provide significant amounts of water for recharge compared to subsurface melt of the Anuchin Glacier. Several studies have already described the structure and function of the microbial communities within the water column and benthic environments of Lake Untersee, however, thus far there have been no studies that examine the linkages between the lake ecosystem with that of the surrounding soils or the Anuchin Glacier. The glacier may also play an important role as a major contributor of nutrients and biota into the lake ecosystem. Based on microbial 16S rRNA amplicon sequencing, we showed that the dominant bacterial signatures in Lake Untersee, the Anuchin Glacier and its surrounding soils were affiliated with *Actinobacteria*, *Bacteroidetes*, *Cyanobacteria*, *Firmicutes*, and *Proteobacteria*. Aerosol and local soil depositions on the glacier surface resulted in distinct microbial communities developing in glacier ice and cryoconite holes. Based on a source tracking algorithm, we found that cryoconite microbial assemblages were a potential source of organisms, explaining up to 36% of benthic microbial mat communities in the lake. However, the major biotic sources for the lake ecosystem are still unknown, illustrating the possible importance of englacial and subglacial zones. The Anuchin Glacier may be considered as a vector in a biological sense for the bacterial colonization of the perennially ice-covered Lake Untersee. However, despite a thick perennial ice cover, observed “lift-off” microbial mats escaping the lake make a bidirectional transfer of biota plausible. Hence, there is an exchange of biota between Lake Untersee and connective habitats possible despite the apparent sealing by a perennial ice cover and the absence of moat areas during austral summer.

## Introduction

The largest ice sheet on Earth covers more than 99.8% of continental Antarctica (Burton-Johnson et al., 2016). Here, the combination of strong winds, low temperatures, high radiation and low water availability are considered to be adverse to life (Boyd et al., 2013). However, there exist a range of habitats with distinct environmental conditions that host microbial dominated communities with active metabolism (Obbels et al., 2016).

The most prominent habitat on glacier surfaces is cryoconite holes that cover 1 to 10% of glacial ablation zones (Anesio and Laybourn-Parry, 2012). They form when dark organic and inorganic debris from local and distant sources attach to the ice surface and consequently decrease the albedo locally (Franzetti et al., 2017). Subsequently, debris melts into the ice, creating basins with sediments in the bottom and a liquid phase on top (Wharton et al., 1985; Takeuchi et al., 2001). Deposited biota that survives local conditions in these mini lakes proliferate while other organisms perish due to stress and competition (Anesio and Laybourn-Parry, 2012). In contrast to cryoconite holes in other regions, the majority of those in Antarctica are continuously ice-covered (Fountain et al., 2004; Cook et al., 2016) and solar-induced subsurface melt provides liquid water only for a mere fraction of the year. Further, they are disconnected from the atmosphere for most of the time, leading to unique environmental conditions such as extremely limited light intensities due to the ice lid (Bagshaw et al., 2016) or unusual hydrochemistry (Fountain et al., 2004). In addition to the habitats provided by cryoconite holes, liquid veins and thin layers of water coating dust grains support microbial life within the glacial ice matrix (Price, 2007). However, compared to cryoconite holes, microbial abundance (Boetius et al., 2015) and metabolic activity (Edwards et al., 2013a) in glacier ice are low.

Glaciers and ice sheets are hydrologically connected ecosystems that constantly receive abiotic and biotic matter and subsequently export material to adjacent downstream environments (Boetius et al., 2015). Therefore, glaciers should not be considered as stand-alone ecosystems but rather as an integral part of large-scale ecosystems. Consecutive habitats such as glaciers melting into lakes, influence their geochemical and microbial composition (Hotaling et al., 2017).

In many cases, Antarctic glaciers are the only obvious hydrological connection to adjacent perennially ice-covered lakes and hence the only source of nutrients and biota (Priscu et al., 1999). Besides subglacial and englacial microbial communities, also those from supraglacial zones may drain into an adjacent waterbody and therefore enable an indirect transfer of organic and inorganic matter from dust and aerosols into downstream aquatic ecosystems via glacial melt. Pre-adapted microorganisms may proliferate efficiently once being transferred to their sink environment. Such pre-adaptation processes may occur in actively metabolizing prokaryotes in the atmosphere (Amato et al., 2017), e.g., in super cooled cloud droplets (Sattler et al., 2001). In addition, aerial input of short and long-range transport are inoculation sources for cold habitats and near surface aerosol microbial communities differ from those within the soils (Bottos et al., 2014).

Here, the perennially ice-covered Lake Untersee is dammed by the Anuchin Glacier that gradually drains into the lake. Among others, studies at the Untersee Oasis included biogeochemical analyses of the water column (Wand et al., 1997, 2006; Haendel et al., 2011), microbial community analyses of benthic (Andersen et al., 2011; Koo et al., 2017b) and pelagic (Filippova et al., 2013; Fomenkov et al., 2017; Pikuta et al., 2017) habitats, and modeling of lake water circulation patterns (Steel et al., 2015). Further, long-term local climate conditions were recorded (Andersen et al., 2015). The research focus during the last decades was set on this lake since the perennial lake ice cover continuously disconnected the waterbody from the atmosphere for at least 500 years (Wand and Perlt, 1999).

As shown in the case of Lake Untersee, the majority of scientific investigations on specific polar environments have mostly been dealt with on a solitary basis, but not in a connective sense. Examples thereof are scarce (e.g., Diaz et al., 2018). However, to understand the linkages between cold habitats and their inoculation vectors (e.g., by melt processes and aeolian transport), a holistic ecosystem approach needs to be considered.

Here, we hypothesized that in the supraglacial zone (a) cryoconite hole microbial communities were dominated by microbial assemblages found in aerosols and adjacent soils and therefore differed from communities originating from bare glacier ice. Despite the presumably small volumetric fraction of cryoconite to the annual glacial melt water input, (b) cryoconite microbial communities can be mirrored in Lake Untersee environments. Biotic fluxes from the Anuchin Glacier to Lake Untersee were put in context by estimating the annual glacial melt volume and the volumetric fraction of cryoconite holes thereof.

### Study Site

**FIGURE 1:**
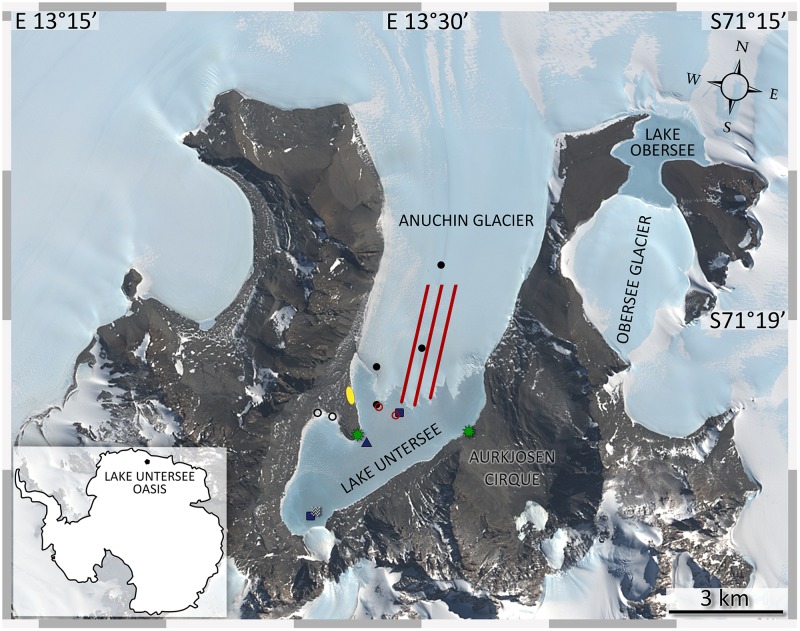
Sampling sites at Lake Untersee Oasis. The image was captured by the Landsat 8 satellite (download via LIMA database from United States Geographical Survey) and color bands 2, 3, 4 were applied to the high-resolution band 8. Lake Untersee is dammed in the north by the Anuchin Glacier. Black dots (cryoconite holes), blue squares (lake sediments), blue triangle (benthic microbial mats), black circles (terrestrial samples), red lines (glacier ice transect, AT1-AT2-AT3 from left to right), red circles (white ice in lake ice cover and lake ice), and a white wave symbol (pelagic samples) indicate the sampling sites. Meteorological stations are indicated by green stars. The yellow ellipse marks the campsite. Overlapping symbols indicate that the sampling site for different sample types was identical.

The Untersee Oasis is part of the Wohlthat Mountains, located nearby the Schirmacher Oasis in East Antarctica (71.348°S, 13.458°E, Figure 1). The area has been ice-free since the Last Glacial Maximum (Hiller et al., 1988), features the largest surface lake in the surrounding which formed during the Holocene (Schwab, 1998) and has no marine origin. The ultra-oligotrophic Lake Untersee located at 610 m above sea level (REMA dataset; Howat et al., 2018) has been continuously ice-covered for at least 500 years (Wand and Perlt, 1999) and is dammed at the northern tip by the Anuchin Glacier with a ∼0.68 km^2^ large ice wall (Steel et al., 2015) that provides the only known liquid water and nutrient source for the lake ecosystem. Water-loss only occurs through sublimation of the perennial lake ice cover (Hermichen et al., 1985). Ablation of ∼60 cm a^-1^ ice thickness during the austral summer is compensated by refreezing of lake water at the bottom of the ice cover during the winter months (Wand and Perlt, 1999). The lake has a 169 m deep basin adjacent to the glacier. Here, the pH reaches values up to 12.1 (Wand et al., 2006). The water column is well mixed, oxygenated and pigmented microbial mats were found up to a depth of 100 m. In shallower waters, modern large conical stromatolites and other benthic microbial mats were discovered (Andersen et al., 2011). These formations are unique to Lake Untersee. Further, a smaller anoxic sub-basin in the southern part of the lake is separated by sill and is physically and chemically stratified from 50 m to the bottom (100 m). Here, high methane concentrations (>20 mmol l^-1^) build up due to methanogenesis in surface sediments and the water column (Wand et al., 2006).

The Anuchin Glacier covers about 34 km^2^ (length 8 km, width 4.2 ± 1.2 km) and drains into the Lake Untersee oxic basin. The surface velocity of the glacier is about 9 m a^-1^ (Wand and Perlt, 1999). This rate was also confirmed by interferometric synthetic-aperture radar data (Rignot et al., 2014), accessed via the QGIS Quantarctica package provided by Norwegian Polar Institute.

The adjacent Lake Obersee (3.4 km^2^, Figure 1) is located 10 km NNE from Lake Untersee and is also dammed by glaciers on the southern and northern side. Surface melt channels from Obersee to Lake Untersee were not observed but are assumed to exist (Simonov et al., 1985).

Based on a 5-years recording from a Campbell meteorological station at the Lake Untersee shore, the mean annual temperature during the austral summer seasons was -10.6°C ± 0.6°C. The most extreme temperatures throughout the year were +9°C (January 2013) and -35.2°C (July 2012). The number of days above freezing ranged from 7 to 49 days. The prevailing wind direction was south with an average daily maximum of 15 m s^-1^ due to katabatic winds from the Wegener ice shelf into the Oasis. The maximum recorded wind speed was 35.7 m s^-1^ (Andersen et al., 2015).

## Materials and Methods

### Meteorological Records

During the 2015 summer field season, an additional custom-built weather station recorded wind speed, direction and temperature with a high temporal resolution (1 min interval, 27266 data points). The station was set up in the Aurkjosen Cirque, at the north-western shore of Lake Untersee from November 25, 2015 until December 14, 2015 (Figure 1).

### Anuchin Glacier Retreat at Surface and Melt Rate Estimation

Two aerial images (from 1939 and 1995, provided by the German Federal Agency for Cartography and Geodesy) and a satellite image from 2015 provided by Digital Globe were imported as layers into Adobe Photoshop and subsequently transformed to account for different angles during image acquisition. Then, the scene was cropped to unify all image-excerpts and the glacier-lake boundaries from each image were mapped into evenly spaced data points with defined x and y pixel coordinates using the software Fiji (Schindelin et al., 2012). The y coordinates from each image were averaged and the resulting differences in pixel location relative to the 1939 position were calculated. The pixel scale was set by counting the number of pixels from geographical features with known dimensions. The length of the glacier-lake boundary for each image was measured with the free-hand line tool in Fiji.

The melt rate of the glacier into the lake was estimated by combining data from a bathymetric map (Wand et al., 2006) and the aforementioned calculation of the glacier retreat. Steel et al. (2015) assumed that the glacier forms a straight wall to the lakebed along the glacier-lake boundary. However, we assumed that the glacier-lake boundary is neither a straight wall nor exactly the shape that can be seen at the surface. Hence, our estimates are averaged values from both scenarios.

### Cryoconite Hole Coverage

Images of the glacier surface were recorded with a drone (DJI phantom 2 with 3-axis stabilized gimbal and GoPro Hero 3+) during two flights between 17:00 and 18:00 UTC. In total, 19 images with a resolution of 12 megapixel were analyzed from the medial moraine (*n* = 10), north of the glacier/lake ice ridge (*n* = 6) and white ice patch within the lake ice (*n* = 3). Batch modifications of all histograms with the software Fiji (Schindelin et al., 2012) improved the contrast between cryoconite holes and glacier ice. Also, dark-colored moraine ice was successfully discriminated from cryoconite holes. Next, the images were thresholded which resulted in region of interests (ROIs) with a variety of shapes. Only ROIs with a roundness > 0.6 were considered in the final calculation where roundness was defined as *4* × Area / (π + Major Axis^2^).

### Sampling

Sampling took place during expeditions to Lake Untersee in November and December 2008, 2014, and in 2015. All sampling locations depicted in Figure 1, 2 were recorded with a handheld GPS (Garmin GPS 60). To follow potential microbial sources and sinks, we took the following connecting sample types of various origins into account: near-ground air as a source for bioaerosols, glacial ice consisting of cryoconite holes and clear ice, lake ice, lake water, lake sediments as well as surrounding soils. All samples were put in sterile containers and stored in coolers that were buried in snow pits and covered with highly reflective mylar blankets during the field season. After the expeditions, the coolers were shipped to Cape Town, repacked with dry ice and further sent to the respective laboratories. The samples were stored at -20°C until further use.

**FIGURE 2:**
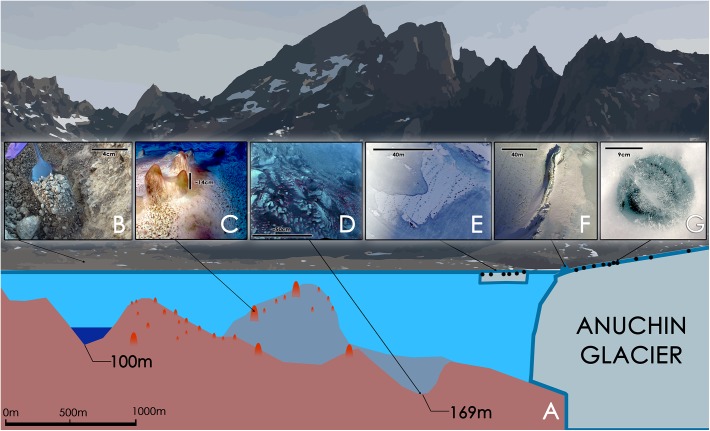
**(A)** Schematic illustration of Lake Untersee with its 169 m oxic basin and a 100 m anoxic basin [illustration based on (Wand et al., 2006)]. **(B)** Soil sample. **(C)** Large conical stromatolites, pinnacle mats and flat mats at a depth of about 24 m. The scale bar is an estimate. **(D)** Pigmented microbial benthic mats at a depth of 169 m. The scale is an estimate. The pink dots are artifacts that were introduced during a white balance correction. They may indicate a similar surface color as seen in **C**. **(E)** “White Ice Patch” – glacial ice integrated in the lake ice containing cryoconite holes that are aligned from NE to SW; the image is oriented with North toward the top of the image. **(F)** Aerial image of a pressure ridge formed by the Anuchin Glacier (left) at the glacier-lake ice interface. Cryoconite holes are visible as dark dots in the glacier ice. The top left corner is orientated toward north. **(G)** Typical morphology of an Antarctic ice-lidded cryoconite hole.

#### Cryoconite Holes

In total, 14 cryoconite holes were collected for this study. Sediments deeper than ∼5 cm were collected with a Kovacs ice corer that was driven by a Bosch Hammer GBH 36V-LI PLUS electrical drill. The core barrel was rinsed with 70% (v/v) ethanol and cleaned with an extra ice core that was drilled next to the cryoconite hole to be discarded. Sediments less than 5 cm deep were collected with a sterile spatula after the ice lid was removed with a sterilized ice axe. Cryoconite hole samples were distinguished into two regions, namely “medial moraine” at the upper part of the glacier medial moraine and “glacier terminus” in proximity of the glacier-lake boundary.

#### Glacial and Lake Ice Samples

We sampled glacial ice along the N-S orientated medial moraine (AT_1) and collected additional transects in clean ice 150 m west (AT_2) and east (AT_3) from AT_1. Each transect consisted of six transect points. The four sampling points closest to the lake were in a distance of 500 m to each other and the upper two sites were kept each at 1000 m to the next point.

At each sampling site, three ice cores from the upper 20 cm of ice were collected with a Kovacs ice corer powered by a 2-cycle Tecumseh motor (2014) or a Bosch Hammer GBH 36V-LI PLUS electrical drill with 4 Ah batteries (2015). A custom-made drill guidance system enabled a standardized spatial sampling of triplicate ice cores due to a rotational axis that kept the distance between the ice samples at fixed levels (Supplementary Figure 1).

Additionally, we sampled two one-meter long ice cores from the Lake Untersee ice cover and the so-called “White Ice Patch” which are further referred to as “US” and “WIP,” respectively. WIP appears to be glacial ice but is part of the lake ice-cover, south of an ice ridge that separated the glacier from the lake at the surface (Figure 2E,F). Each sample of the 1 m long ice core was 33 cm long and categorized as top-middle-bottom sections.

For all drilled samples, post-sampling decontamination procedures (Legrand and Mayewski, 1997; Christner et al., 2005; Doyle et al., 2013) could not be applied due to the brittleness of the ice cores. Instead, a thorough pre-sampling cleaning approach was established: The sampling site was always approached from the leeward side, the core barrel was cleaned with 70% (v/v) ethanol and subsequently flushed with an original ice sample to be discarded afterward.

#### Pelagic and Benthic Lake Samples

Samples of lake water at the anoxic basin were collected in 2008 at 8, 30, 40, 50, and 70 m with a Niskin bottle. Water samples were kept frozen throughout storage in the field and subsequent transport to the home university with dry ice. Lake sediments from the oxic (169 m) and anoxic (100 m) basins were collected with an Ekman dredge connected to a parachute cord. The samples were transported to the campsite and immediately transferred into sterile bags using a baked spatula. Other benthic samples, namely flat mats and pinnacle mats were collected by a scuba diver at a depth of ∼30 m in the oxic basin using methods as described by Andersen et al. (2011).

#### Soils

Soil samples were collected with a baked spatula at the south-western shore of the lake. They were associated with a microbial mat, buried 3 cm deep in gravel and a gray crust on a rock.

#### Bioaerosols

Samples of near-ground air were collected in 2015, at the windward side of the campsite to avoid contamination from the camp itself. Airborne particles were accumulated in a sterile sodium chloride solution (0.9%) with a Bertin Coriolis-μ air sampler. To prevent the liquid from freezing during the sampling procedure, the sampling container was placed in a Nalgene bottle with hot water. After sample collection, the sampling containers were stored with previously collected ice cores that served as a cooling agent. In total, seven samples have been collected over a period of 3 days with a flow rate of 300 L^-min^ ranging from 3,000 to 12,000 liters per sample, yielding to 45,000 L total volume. The captured airborne particles had to be pooled into one sample to gain sufficient DNA content.

### Microbial Analysis

#### Sample Processing and DNA Isolation

All samples were thawed thoroughly in the dark at 4°C. DNA from benthic microbial mats, terrestrial samples and a cryoconite holes was extracted using the PowerSoil^®^DNA Isolation Kit. PowerWater^®^DNA Isolation Kit was used for the bioaerosols, ice and water samples following their respective manual’s instructions. The DNA content was measured with a Qubit^TM^ dsDNA HS Assay Kit before 10 ng DNA of each sample were applied as template in subsequent PCR reactions.

#### Amplicon Generation for Next Generation Sequencing (NGS)

The 16S rRNA gene amplicons for sequencing were obtained using Illumina-tagged universal primers F515 and R806 as proposed by the Human Microbiome Project and the Earth Microbiome Project (Caporaso et al., 2012) and archaea- targeting primers as proposed by Klindworth et al. (2013). For this approach, we selected the protocol as already proposed by Koskinen et al. (2017) which is based on a nested PCR approach using primers 344f and 915r in the first PCR and primer pair S-D-Arch-0519-a-S-15/S-D-Bact-0785-b-A-18 in the subsequent PCR. More information on cycling condition and primer sequences can be found in Koskinen et al. (2017). Library preparation and MiSeq sequencing were carried out at the Core Facility Molecular Biology at the Center for Medical Research, Graz, Austria (Klymiuk et al., 2016).

#### Next Generation Sequence Analysis

Raw sequences were pre-processed and filtered using the R package dada2 (version 1.4.0) according to the proposed processing pipeline (Callahan et al., 2016). Briefly, reads were demultiplexed, forward and reverse reads were quality filtered (min. score: 30), merged and the dada2 core algorithm was applied. The taxonomy was assigned to the SILVA 123 database (Quast et al., 2013) and a RSV table was generated. A more detailed workflow can be found in Mora et al. (2016). The downstream analysis was generated using R (version 1.0.36) as described in the following.

#### Statistical Analysis

The RSV table (ribosomal sequence variants) was used to calculate alpha and beta diversity using the R packages Phyloseq (McMurdie and Holmes, 2013) version 1.20.0. To compare microbial diversity in different Lake Untersee environments (oxic and anoxic sediments, benthic microbial mats and pelagic samples), alpha diversity was calculated using the Shannon, Observed and Inverse Simpson index. As discussed in McMurdie and Holmes (2014), rarefying counts may result in a high rate of false positives that are differently abundant across samples and thus, no rarefaction was applied. Normal distribution was tested by the Shapiro–Wilk test and significances were tested either by Kruskal–Wallis (if not normal distributed) or an ANOVA (if normal distributed). The Dunn’s test was used as *post hoc* test (Bonferroni corrected). Analysis of beta diversity was performed to examine differences between microbial community structures in Lake Untersee environments. Principal Coordinates Analysis (PCoA) was performed directly on the (to relative abundance transformed) RSV table. Significances were tested using adonis (seed 1) and further confirmed by dispersion tests (permutations: 999). Source tracking was performed using the sourcetracker algorithm as proposed by Knights et al. (2011) and the Venn diagram was generated using the web-based tool InteractiVenn (Heberle et al., 2015).

#### Negative Controls and Data Storage

Every step of the analysis included negative controls consisting of blank extractions for each group of samples and negative controls during PCR amplifications. RSVs that overlapped negative controls and samples were removed from the data sets. Sequence data have been deposited in the NCBI database under the accession number SRP145579.

## Results

### Glacier Retreat, Melt Rate and Cryoconite Hole Coverage

**FIGURE 3:**
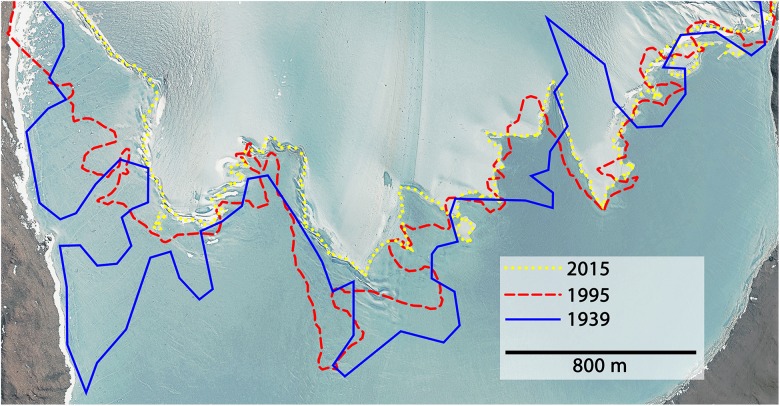
The glacier-lake boundaries are shown for the years 1939 (blue), 1995 (red), and 2015 (yellow). Satellite image © DigitalGlobe, Inc. Provided by NGA Commercial Imagery Program.

The average retreat within 76 years (1939–2015) was 2.37 m a^-1^. During the first 56 years (1939–1995) the glacier retreated on average by 1.16 m a^-1^ and increased up to 5.76 m a^-1^ during the last 20 years (1995–2015). Combining these calculations with data from a bathymetric map of Lake Untersee (Wand et al., 2006), the melt water contribution to the lake within 76 years was 7.83 × 10^7^± 2.07 × 10^7^mł which equaled an average melt rate of 1.03 × 10^6^± 2.72 × 10^5^mł a^-1^. Between 1995 and 2015, the rate increased 2.34-fold compared to the long-term average, meaning that 63.9% of the total melts happened during the last quarter of this 76-year period. The long-term annual melt input corresponded to 1.18 × 10^-1^± 3.12 × 10^-2^% of the current estimated lake volume (0.872 kmł). Further, the length of the surface glacier-lake boundary smoothened and therefore decreased by 2.85 km. About 82% of this reduction happened between 1995 and 2015. The position of the Anuchin Glacier for the years 1939, 1995, and 2015 is depicted in Figure 3.

The cryoconite holes coverage on the Anuchin Glacier was 3.46 ± 2.34% on average. The highest density was observed near the glacier-lake interface at the SW part of the glacier (6.36 ± 0.98%) and the lowest coverage was observed along the medial moraine (1.73 ± 0.36%).

Assuming an average cryoconite layer thickness of 1 cm, we estimated that the long-term (1939–2015) average contribution to the lake ranged between 3.69 and 13.56 m a^-1^ and from 8.96 to 32.94 m a^-1^ in recent years (1995–2015), respectively. The maximum volumetric portion of cryoconite to the annual melt was 1.32 × 10^-3^% on average within 76 years, representing 1.56 × 10^-6^% of the present estimated lake volume.

### Wind and Temperature Regime at Lake Untersee

About 77% of the time local winds from 90 to 160° were recorded during a 20-day period (Supplementary Figure 2). The highest average wind speeds (1 min interval, 8 m s^-1^) and maximum wind speed (24.6 m s^-1^) were associated with the prevailing wind directions. Average hourly wind speeds above 7 m s^-1^ occurred only between 02:00 and 10:00 and the average temperature was -0.08°C (min. -8.3°C, max. 7.2°C).

### The Bacteriome of Lake Untersee Oasis

For bacterial community profiles, the prevailing information was grouped into seven different habitats: “Air” (air sample), “Terrestrial” (buried mat and crust from a rock), “Pelagic” (water column of Lake Untersee), “Benthic” (benthic microbial mats and sediments from Lake Untersee), “Lake ICE” (lake ice from Lake Untersee), “Glacier ICE” (surface ice of Anuchin Glacier) and “Cryoconite” (cryoconite holes from Anuchin Glacier). Amplicon sequencing resulted in a total of 1,414,290 read counts, including 4,772 taxonomic observations in 52 samples. The amplicon read counts for all samples are depicted in Supplementary Figure 3, ranging from 3,545 counts in sample “Cryoconite 5” to 50,838 counts in sample “WIP_bottom.”

**FIGURE 4:**
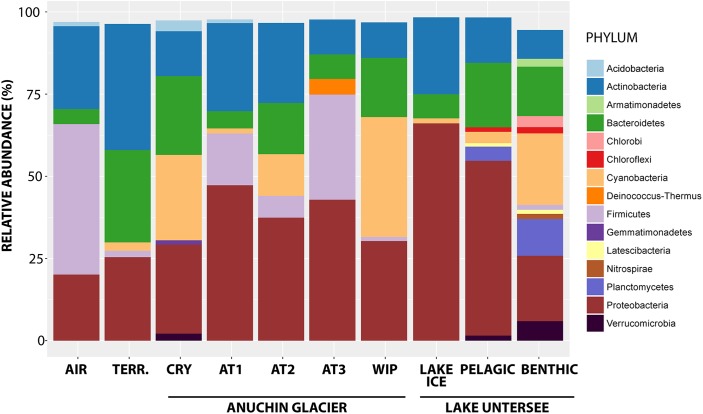
Bacterial phyla from the Anuchin Glacier, Lake Untersee and its surroundings. TERR, terrestrial samples; CRY, cryoconite holes; AT1-3, parallel ice transects along Anuchin Glacier; WIP, white ice patch in Lake Untersee. Sequences unidentified at the genus level and low abundant phyla (abundance < 1%) were removed.

Overall, signatures of 42 bacterial phyla were detected, whereas most of the RSVs were affiliated with *Proteobacteria* (35.4% of all RSVs), followed by *Actinobacteria* (17.9%), *Cyanobacteria* (13%) and *Firmicutes* (7.6%). The highest percentage of reads was assigned to the bacterial genera *Methylobacterium* (1.9% of all RSVs), *Reyranella* (1.7%), *Sphingomonas* (1.8%), *Polaromonas* (1.8%) and *Acinetobacter* (2.4%). *Tychonema* (6.7%), *Chamaesiphon* (2.5%), and *Leptolyngbya* (2.7%) comprised most affiliated reads for the phylum *Cyanobacteria*. An overview of the most abundant phyla across the sampling sites is depicted in Figure 4.

**FIGURE 5:**
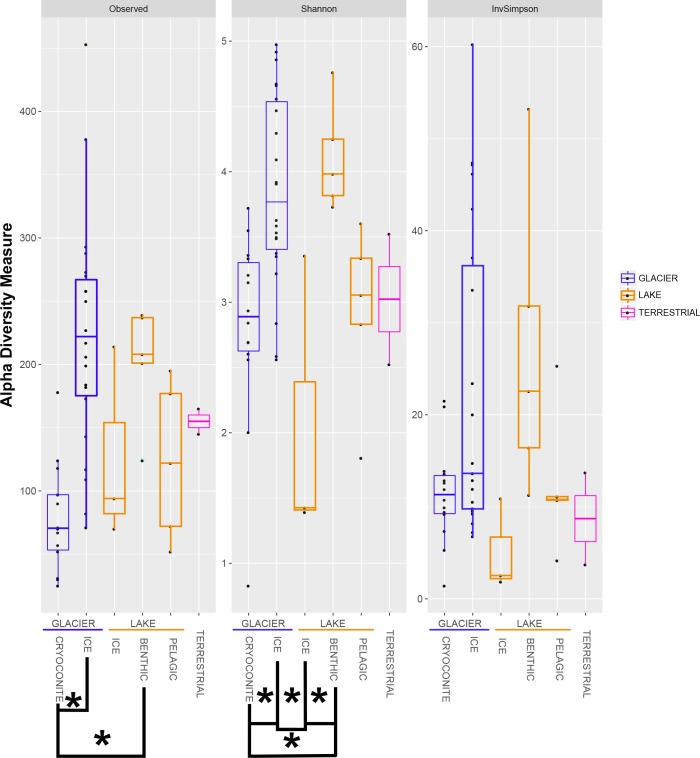
The alpha diversity indices Observed, Shannon and InvSimpson are shown in boxplots. All samples are grouped into similar habitats. Significant differences are marked with clamps and stars.

The bacterial alpha diversity was calculated using the indices Observed, Shannon and InvSimpson and is depicted in a boxplot (Figure 5). Data for the Observed and InvSimpson indices were not normally distributed (Shapiro–Wilk Test: *p* < 0.05) and differed significantly (*p* = 1.078 × 10^-4^ and 0.04, respectively; Kruskal–Wallis Test). This was also true for the normally distributed dataset observed for the Shannon index (*p* = 6.68 × 10^-5^; ANOVA).

There were significant differences between the groups “GLACIER ICE” / “GLACIER CRYOCONITE” and “GLACIER ICE” / “LAKE BENTHIC” when using the Observed index (*p* = 3.261232 × 10^-5^ and 4.102203 × 10^-2^, respectively; Dunn’s Test, the *p*-values were adjusted using the Bonferroni method). In total, 4 groups were significantly different when observing the Shannon Index (“GLACIER ICE” / “GLACIER CRYOCONITE” *p* = 0.001; “LAKE BENTHIC” / “LAKE ICE” *p* = 0.004; “LAKE BENTHIC” / “GLACIER CRYOCONITE” *p* = 0.017; “LAKE ICE” / “GLACIER ICE” *p* = 0.002; Tukey’s *post hoc* test, *p*-values were adjusted using Bonferroni). The pooled air sample had to be excluded from statistical analysis.

**FIGURE 6:**
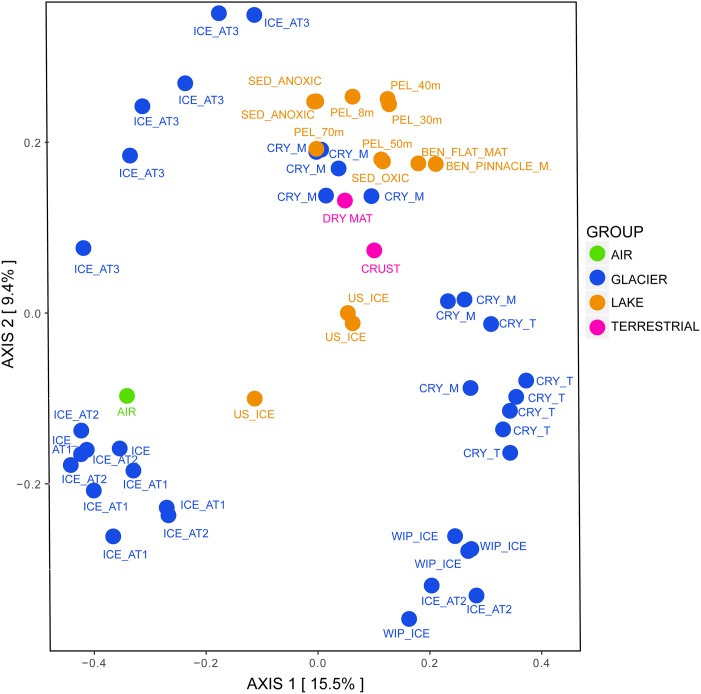
Bacterial beta diversity in the Untersee Oasis displayed as PCoA plot using Bray-Curtis distance matrix. The habitats “Air,” “Anuchin” (glacier), “Lake” (Untersee), and “Terrestrial” are color coded. CRY_M, Cryoconite holes from the medial moraine; CRY_T, Cryoconite holes from the glacier terminus; SED, Sediment; PEL, Pelagic; BEN, Benthic. Further differentiation is described in the Section “Materials and Methods.”

Regarding the beta diversity, the samples clustered according to their grouping and were significantly different from each other (Figure 6, ANOSIM: 999 permutations, *p* < 0.001). Most of cryoconite holes from the medial moraine overlapped with lake samples while cryoconite holes from the glacier terminus were rather isolated from other lake samples. Except for the lake ice cover, lake samples also clustered with terrestrial samples. The glacier ice formed three distinct clusters; two of them were associated with white ice patch samples and one with the air sample, respectively.

**FIGURE 7:**
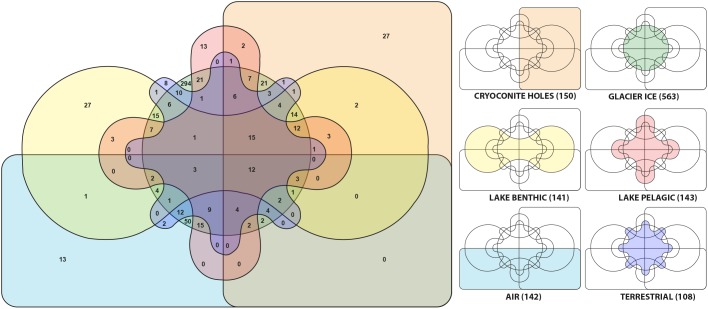
Genera from six different habitats are displayed in a Venn-diagram. For clarification, the colors and positions of each habitat are separately shown as mini diagrams. The numbers in brackets indicate the number of genera at each location.

We observed 12 out of 1247 genera (0.96%) that occurred in all habitats. They were affiliated with *Tychonema*, *Leptolyngbya*, *Opitutus*, *Gemmatimonas*, *Brevundimonas*, *Pedobacter*, *Cryobacterium*, *Flavobacterium*, *Gemmata*, *Roseomonas*, *Blastocatella*, and *Novosphingobium*. Microbial communities in aerosols harbored 142 genera and shared 96 of them (67.6%) with glacier ice communities and 79 (55.6%) with cryoconite holes, respectively. The supraglacial environment contained in total 713 genera, 112 of them (10.55%) were present in both, glacial ice and cryoconite holes while 38 genera (3.58%) were exclusive to cryoconite holes and 563 genera (42.47%) were only found in glacial ice. From 108 OTU’s that were identified in terrestrial sources 36 (33.3%) of them were also discovered in cryoconite holes, glacial ice and the lake. The lake harbored 284 different genera. Lake sediments and the water column shared 62 (21.8%) of them. In the lake and on the glacier, 177 genera (21.5% of genera affiliated with the lake) were identical with glacier affiliated genera (Figure 7).

We focused on the supraglacial habitats cryoconite holes and glacial ice as they were different from each other in richness and diversity. Taxa with significantly different abundances were determined using the Mann–Whitney test (with FDR – false discovery rate correction for multiple comparisons). In total, 92 RSVs were significantly different abundant (*p* < 0.05). The majority were abundant in the glacier ice but not abundant in cryoconite holes. Most of the RSVs could not be assigned to any known taxon.

**FIGURE 8:**
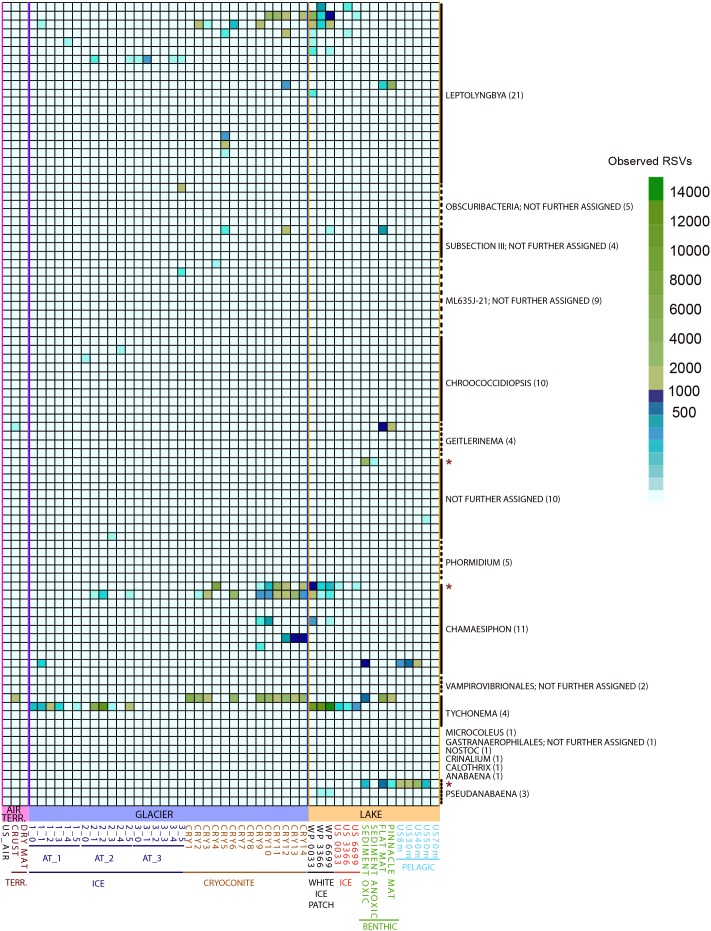
Observed RSVs belonging to the phylum *Cyanobacteria* (affiliated to genus level, numbers of RSVs in brackets) in individual samples, depicted as heatmap. Significantly different abundant RSVs are marked with a red star.

Since cyanobacteria merit special emphasis in cryospheric habitats (Vincent, 2000; Namsaraev et al., 2010; Chrismas et al., 2015) we counted RSVs assigned to this phylum and tested for significant differences in abundance for the groups “GLACIER ICE,” “GLACIER CRYOCONITE,” “LAKE BENTHIC,” “LAKE PELAGIC,” and “Lake ICE” (Figure 8). The cyanobacterial abundance accounted for 13% in the entire bacteriome with the highest presence in the groups “GLACIER CRYOCONITE” (25.4% of the overall cyanobacterial affiliated RSV counts), followed by the group “LAKE BENTHIC” (21.4%) and “GLACIER ICE” (13.6%). Fewer cyanobacterial affiliated RSVs were detected in the samples “LAKE PELAGIC” (3.4%), TERRESTRIAL (2.6%) and “AIR” (0.9%).

Three genera were significantly different abundant: *Chamaesiphon*, *Pseudanabaena* and RSV belonging to an unidentified genus (Mann–Whitney *U* Test, FDR corrected, *p* < 0.05). Here, the highest amount of genus-assigned RSV counts was affiliated to *Chamaesiphon* in the group “GLACIER CRYOCONITE” (9.8% of all cyanobacterial affiliated RSV counts) and *Pseudanabaena* in the group “LAKE PELAGIC” (2.5%).

### The Archaeome of Lake Untersee Oasis

In total, 13 samples did not comprise any RSV affiliated to archaea (amongst them the “AIR” sample and most of the “LAKE ICE” samples). From all other samples, 185,004 archaeal RSV counts were obtained. Exclusively, all RSVs were affiliated to *Euryarchaeota* and the highest amount of all archaeal RSV counts was classified as *Methanomicrobia* (51.8%), followed by *Halobacteria* (43.2%) and low numbers of *Thermoplasmata* (>5%), *Methanobacteria* and *Thaumarchaeota* (both > 1%).

**FIGURE 9:**
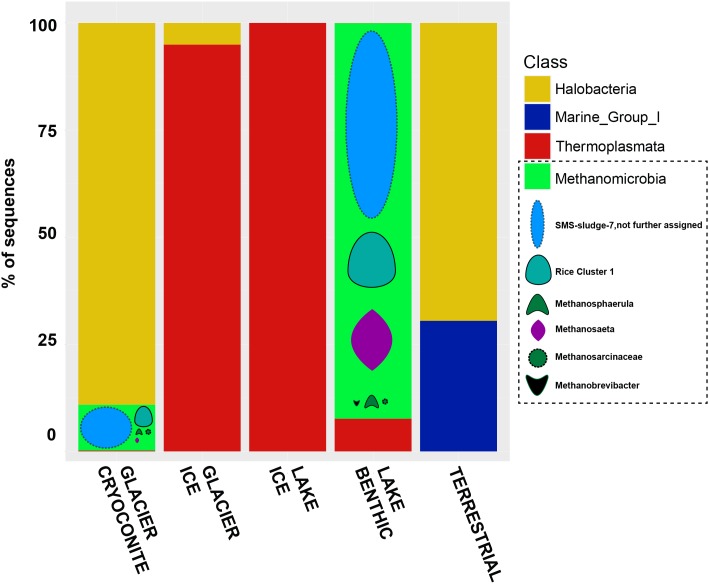
Bar chart of archaeal sequences assigned to their respective habitats. *Methanomicrobia* are further classified into the groups *SMS-sludge-7*, *Rice Cluster 1*, *Methanospaerula*, *Methanosaeta*, *Methanosarcinaceae*, and *Methanobrevibacter*.

Methanogens were exclusively detected in the groups “LAKE ICE” and “LAKE BENTHIC” as well as a high majority of unassigned *Methanobacteriales-SMS-sludge 7* (almost 70% of all methanogen-related affiliated RSV counts). *Methanosaeta* (almost 16%) and *Methanocellaceae* (Rice Cluster 1, almost 12%) comprised also a high RSV count, while low abundant RSVs were affiliated to *Methanobrevibacter* and *Methanosarcina* (<1%) (Figure 9).

The archaeal alpha diversity was calculated as described in the section “bacterial alpha diversity.” The highest diversity and richness were detected in the group “LAKE BENTHIC,” followed by “GLACIER ICE” and “GLACIER CRYOCONITE.” The groups “TERRESTRIAL” and “LAKE ICE” comprised a very low diversity and almost all samples within these groups did not contain any Archaea at all (Supplementary Figure 4). Statistical analyses as performed for the bacterial dataset were not applicable for the archaeal dataset. The low abundance and/or absence of archaeal related RSVs in many samples violated the conditions for these analyses.

**FIGURE 10:**
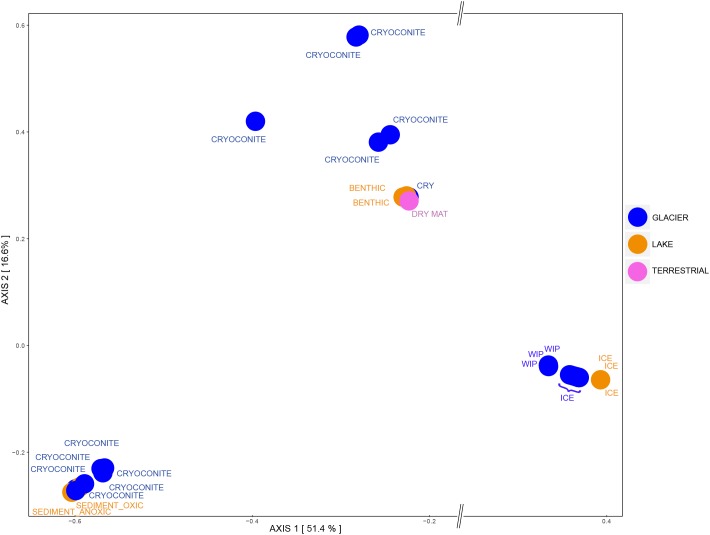
Archaeal beta diversity at Lake Untersee Oasis. Habitats are color-coded and further description is depicted as text: CRY, Cryoconite holes; WIP, White ice patch. For a better visualization, an axis break on the *x*-axis was introduced.

Principle Coordinates Analysis showed that samples that contained archaeal sequences formed three separate clusters. All ice samples (“GLACIER ICE” and “LAKE ICE”) lined up in the same area and showed the largest distance on the *x*-axis (51.4%) to other assemblages, namely “GLACIER CRYOCONITE” and lake sediments from the group “LAKE BENTHIC.” The remaining “ANUCHIN CRYOCONITE,” “TERRESTRIAL,” and “LAKE BENTHIC” samples formed a third cluster (Figure 10). An analysis of similarity as done for the bacterial beta diversity was not applicable for the archaeal dataset due to the absence of Archaea in too many samples.

### Sourcetracking

**FIGURE 11:**
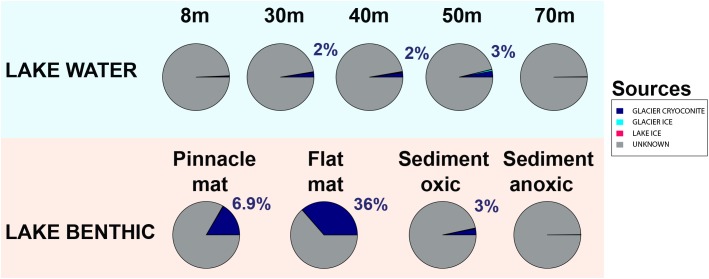
Defining potential sources of the microbial community using the Bayesian community-wide culture-independent microbial source tracking algorithm. Most source environments remained unknown (depicted in gray). Most of the known source environments belonged to the group “GLACIER CRYOCONITE.”

To estimate source environments for the microbial distribution, we applied a source tracking algorithm (Knights et al., 2011). Geographically, Lake Untersee habitats “LAKE BENTHIC” and “LAKE PELAGIC” represented the “sink.” Almost all configurations in this estimate were not assigned to a known source location (Figure 11). However, the location “GLACIER CRYOCONITE” was identified as the inoculation source of highest impact amongst all known locations, contributing up to 36% to the sink environment.

## Discussion

### Biotic Transfer From the Anuchin Glacier to Lake Untersee

A diverse range of habitat types at the Lake Untersee Oasis host microbial life including glacier surfaces, ice-free areas, and a perennially ice-covered lake with its pelagic and benthic zones. These refugia are geographically connected as aerial and local depositions inoculate cryoconite holes on the glacier surface and subsequently colonize Lake Untersee. Generally, extremely short periods in the year provide subsurface melt water within cryoconite holes that ensure biotic exchange and the surrounding glacial ice. However, sub-surface glacial melt into Lake Untersee exists throughout the year with sub-annual varying rates (Steel et al., 2015).

We used a Bayesian source tracking algorithm (Knights et al., 2011) to estimate the proportion of source habitats (supraglacial ice, cryoconite holes, soils, aerosols) to a sink environment (benthic and pelagic zones within Lake Untersee) with potential unknown sources such as englacial and subglacial zones. We showed that the proportion of cryoconite holes as source for benthic microbial mats in Lake Untersee made up to 36% while all other sampled source habitats as such were negligible (Figure 11). Considering that the estimated average annual volumetric contribution of cryoconite to the total melt volume was 1.13 × 10^-3^%, the proportion of cryoconite hole communities as source environment appeared to be substantial.

Most biotic sources that inoculated Lake Untersee benthic and pelagic zones were not identified in this study and the proportion of known source environments differed across the sink samples. We expected biota entering the lake being present in the water column and benthic zones but pelagic microbial communities did not appear as an important sink environment. Considering that the lake was not permanently ice covered (Schwab, 1998) until at least 500 years ago (Wand and Perlt, 1999), it is likely that it was primarily colonized before the formation of today’s perennial ice cover. Hence, a limited biotic transfer into the lake caused by the presence of an ice cover at the time of sampling would explain why the waterbody was not classified as an important sink environment. Also, our sampling efforts only allowed a temporal snapshot of the microbiome of Lake Untersee Oasis. In contrast, high estimates of cryoconite biota as source for the benthic zone may be partly caused by so-called “lift-off” microbial mats that reinoculate the Anuchin Glacier and hence might have the fate of being recycled in this loop. Benthic microbial mats form gas bubbles that increase the buoyancy and therefore disrupt the mats. Consequently, they float upward and integrate into the lake ice cover as the ice thickens from the bottom and resurface as the lake ice cover ablates. Local winds measured by Andersen et al. (2015) and during our expedition (Supplementary Figure 2) would predominantly disperse these mats from the lake surface toward the Anuchin Glacier. This lift-off effect was observed at Lake Untersee and is also known from other Antarctic perennially ice-covered lakes (Parker et al., 1982; Moorhead et al., 2003). However, it is not clear whether this mode of dispersal played a significant role at our study site.

### Microbial Communities in Source Habitats for Lake Untersee Microbiome

Bioaerosol samples from Lake Untersee Oasis were dominated by *Firmicutes*, *Proteobacteria* and *Actinobacteria*. *Cyanobacteria*, which are known to be deposited as bioaerosols (Michaud et al., 2012), accounted only for 0.9% of the community. In comparison, bioaerosols from the Antarctic Miers Valley were also dominated by *Firmicutes* followed by *Acidobacteria*, *Planctomycetes* and *Actinobacteria* (Bottos et al., 2014). Further, we found members of the genera *Staphylococcus*, *Bacillus*, *Corynebacterium*, *Micrococcus*, *Streptococcus*, and *Neisseria* that are commonly reported in the literature of Antarctic related and general bioaerosol studies (Cuthbertson and Pearce, 2017).

Fluxes of bioaerosols depend on parameters such as wind speed and direction (Crawford et al., 2017) and vary at a temporal scale (Fierer et al., 2008; Šabacká et al., 2012). At Lake Untersee, the prevailing wind direction and the highest wind derived from the south (Andersen et al., 2015). Also, high wind velocities were measured from the Aurkjosen Cirque where our temporary weather station was set up during the field season 2015. There, the strongest winds and the dominant wind direction were recorded from S-SE (Supplementary Figure 2). Thus, local aerosolized terrestrial sources from the southern part of the Untersee Oasis and from Aurkjosen Cirque are likely to be dispersed on the ice surface of the Anuchin Glacier.

Local wind systems may influence not only microbial communities in cryoconite holes but also induce the formation of these habitats. For instance, a glacier terminating into the southern part of Lake Obersee (Figure 1) lacks cryoconite holes (personal observation). There, the predominant wind direction (presumably from the East) may not promote the allocation of a sufficient amount of local terrestrial sources onto the glacier. There, the catchment area with potential dust sources in the windward direction of the glacier is small, compared to the setting at the Anuchin Glacier. Whether these local wind systems also influence the composition of microbial communities in glacial ice is unknown and requires further investigation.

For soil samples, the dominant phyla from the Untersee Oasis were *Actinobacteria*, *Bacteroidetes*, *Proteobacteria*, *Cyanobacteria*, and *Firmicutes*. In comparison, Dry Valley soil bacterial communities showed a higher diversity, consisting of *Proteobacteria*, *Actinobacteria*, *Acidobacteria*, *Gemmatimonadetes*, *Bacteroidetes*, *Deinococcus–Thermus*, *Cyanobacteria*, *Planctomycetes*, *Chloroflexi*, and less abundant other phyla (Cary et al., 2010).

Aerial dispersal of biota may be one of the main vectors for the inoculation of isolated and remote habitats (Bowman and Deming, 2017). The same assumption was made for cryoconite holes from the McMurdo Dry Valleys because the adjacent surroundings did not reflect the biotic composition in these mini-ecosystems (Porazinska et al., 2004). Also, communities in cryoconite holes and ice-marginal environments from Arctic glaciers (Edwards et al., 2013b) and from a glacier in the Italian Alps (Franzetti et al., 2017) were distinct.

Biotic input from melting ice surrounding cryoconite holes is an underestimated source for cryoconite hole communities as already proposed by Hodson et al. (2013). Cryoconite hole microbial communities from the Untersee Oasis consisted of *Proteobacteria*, *Cyanobacteria*, *Bacteroidetes*, *Actinobacteria*, *Acidobacteria*, *Verrucomicrobia*, and *Gemmatimonadetes*. Communities from the same habitat type at the Taylor Glacier in the McMurdo Dry Valleys harbored the same dominating phyla as we have identified at the Untersee Oasis but their relative abundance varied (Christner et al., 2003). Further, Cameron et al. (2012) emphasized that *Gemmatimonadetes* – related sequences were present in cryoconite holes from the Vestfold hills but absent in samples outside Antarctica, including Greenland, Svalbard and Norway. We also have identified *Gemmatimonadetes* related sequences in 78% of all cryoconite hole samples from a single glacier and Christner et al. (2003) also identified this phylum in Antarctic cryoconite holes from Canada Glacier.

Bare ice is considered as a habitat supporting active microbial life (Edwards and Cameron, 2017). Lake Untersee ice samples were dominated by *Proteobacteria*, *Actinobacteria*, *Bacteroidetes*, and *Cyanobacteria*. In contrast, samples from the white ice patch within the Lake Untersee ice cover were dominated by *Cyanobacteria*, *Proteobacteria* and *Bacteroidetes*. In both types of sediment-free ice samples, the cyanobacterial genera *Tychonema*, *Leptolyngbya* and *Chamaesiphon* were dominant. In contrast, *Cyanobacteria* affiliated with the genera *Phormidium*, *Chamaesiphon*, *Leptolyngbya*, and other taxonomical not assigned *Cyanobacteria* in the sediment rich lake-ice cover of Lake Bonney in the Antarctic Dry Valleys comprised 20% of the microbiota (Priscu et al., 1998).

Surface ice samples from the Anuchin Glacier were divided into three transects, namely the medial moraine (AT1) that was flanked by two parallel transects (AT2, AT3) with clear ice. We found *Proteobacteria*, *Actinobacteria*, *Firmicutes*, *Bacteroidetes*, and *Cyanobacteria* to be dominant in all glacial ice samples. Within the phylum *Proteobacteria*, *Betaproteobacteria* were dominant in the medial moraine (AT1), whereas *Gammaproteobacteria* (AT2) and *Alphaproteobacteria* (AT3) were more abundant in the clean-ice parallel transects.

Genera belonging to *Cyanobacteria* that were present in the lake ice and the white ice patch were also discovered in at least one of the glacier transect points. Within *Cyanobacteria*, *Chroococcidiopsis* was found in AT1 and AT2 while the genera *Geitlerinema*, *Microcoleus*, *Nostoc*, *Phormidium*, and *Pseudanabaena* were mainly present along the medial moraine (AT1), indicating that different genera of *Cyanobacteria* prefer specific local conditions or that biotic sources differ among transects.

Cryoconite holes are considered as microbial hotspots in supraglacial Arctic environments due to a high biodiversity (Edwards et al., 2013a; Cook et al., 2016). Contradictory, bacterial alpha diversity from cryoconite holes at the Anuchin Glacier was significantly lower compared to the surrounding supraglacial ice which had the highest diversity from all Lake Untersee Oasis habitats (Figure 5). The presence of an ice-lid on Antarctic cryoconite holes and hence a limited exchange with adjacent habitats may explain these discrepancies. However, limitation in NGS analysis includes uneven sample depth and full certainty on actual microbial communities cannot be provided. Further analyses need to be carried out in order to gain a more accurate picture.

Microbial assemblages in cryoconite holes and the glacier ice were significantly different (Figure 6). The most relative abundant and significant contributors in cryoconite holes (7.3%) and surface ice (5.65%) were taxonomically not assigned and except for *Actinobacteria* in cryoconite holes and *Thermoleophilia* in glacier ice, less than 50% of the genera within the significantly different classes (*Thermomicrobia*, *Rubrobacteria*, *Negativicutes*, *Gammaproteobacteria*, *Erysipelotrichia*, *Clostridia*, *Betaproteobacteria*, *Bacteroidia*, *Bacilli*, and *Alphaproteobacteria*) could not be assigned either. Cameron et al. (2016) compared supraglacial communities on a larger spatial scale across the Greenland ice sheet and found that the sample type (cryoconite vs. ice) accounted only for 2.3% of the total variability in supraglacial microbial community composition.

**Pelagic microbial communities** from Lake Untersee were dominated by *Proteobacteria*, *Bacteroidetes*, *Actinobacteria*, *Planctomycetes*, and *Cyanobacteria*. In comparison, within three interconnected lakes located in the nearby Schirmacher Oasis, *Chloroflexi* was among the most abundant phyla and another series of connected lakes from the same area was rather dominated by *Firmicutes* than by *Bacteroidetes* (Huang et al., 2014). The authors concluded that each series of lakes can be considered as one large lacustrine ecosystem due to their interconnectivity. Further, Lake Tawani, another waterbody of the Schirmacher Oasis, harbored *Proteobacteria* and *Bacteroidetes* as the other lakes did, but members of the phyla *Verrucomicrobia*, *Candidate Division TM7*, and *Planctomycetes* (Huang et al., 2013) were absent in the interconnected lakes studied by Huang et al. (2014).

We analyzed **benthic microbial mats** from a depth of about 30 m, sediments from both an anoxic basin at 100 m and from an oxic basin with a maximum depth of 169 m. As reported elsewhere (e.g., Andersen et al., 2011; Wilkins et al., 2013; Mackey et al., 2015; Findlay and Battin, 2016; Koo et al., 2017a,b; Tanabe et al., 2017) benthic microbial mats are usually dominated by *Cyanobacteria*. Lake Untersee benthic microbial mat samples (flat mat and pinnacle mat) were no exception from previous observations and harbored *Cyanobacteria*, *Proteobacteria*, *Bacteroidetes*, *Planctomycetes*, *Verrucomicrobia*, and *Actinobacteria*. In contrast, the main phyla in the oxic sediment belonged to *Bacteroidetes*, *Proteobacteria*, *Cyanobacteria*, *Actinobacteria*, and *Planctomycetes*. In the anoxic sediments we found *Proteobacteria*, *Chlorobi*, *Actinobacteria*, *Planctomycetes*, and *Armatimonadetes* as dominating phyla. It is noteworthy that differences in sampling strategies between the benthic microbial mat samples (carefully collected by a scuba diver) and deep lake sediments (Ekman dredge) hampered this comparison. Based on *in situ* images in the oxic basin (Figure 2D), we would have expected that *Cyanobacteria* were more abundant.

Further, *Proteobacteria* were the most or second most abundant phylum in all lake samples (benthic, pelagic, ice). Together with *Bacteroidetes* and *Actinobacteria*, they comprised 99.2% of the bacteriome in Lake Untersee sediments. Koo et al. (2017b) found that the amount of *Cyanobacteria* in three distinct laminae of a benthic microbial mat from Lake Untersee decreased with depth ranging from about 90% (top) to 20% (bottom). In our study, only bulk samples were processed and hence, results are not directly comparable. However, the relative abundance of *Cyanobacteria* within benthic microbial mats in both studies was in the same range.

***Cyanobacteria*** are the most important phototrophs in freshwater and terrestrial ecosystems (Vincent, 2000; Namsaraev et al., 2010; Chrismas et al., 2015) and colonize many habitats in Antarctica (Makhalanyane et al., 2015). The scarcity of vascular plants in continental Antarctica emphasizes the role of these microbial ecosystem engineers. *Cyanobacteria* accounted for 13% of the entire Lake Untersee Oasis bacteriome and the genera *Leptolyngbya* and *Tychonema* were present in all habitats. Generally, the highest abundances of *Cyanobacteria* were found in habitats associated with sediments in combination with high water availability such as cryoconite holes and benthic microbial mats, opposed to bioaerosols, ice and dry soils. Further, the same pattern occurred at RSV level, indicating a link between ribosomal sequence variants and habitat specific conditions (Tikhonov et al., 2014). For example, one *Tychonema* affiliated RSV was abundant only in these sediment-associated zones. The opposite pattern was observed in another RSV from the same genus. Moreover, 22 RSVs of the genus *Leptolyngbya* differed in their abundance within single habitats. For example, both benthic microbial mats harbored one specific *Leptolyngbya* related RSV that was also found in cryoconite hole 12 but the cryoconite sample contained two additional and abundant *Leptolyngbya* affiliated RSVs that were absent in the benthic microbial mats (Figure 8). This micro-heterogeneity within single genera may suggest that microbial communities adapt to habitat-specific conditions and merits deeper insight.

**Archaea** were not present in all samples. All identified RSVs belonged to the phylum *Euryarchaeota* and most of the counts originated from the oxic (55.77%) and anoxic sediments (33.11%), mainly belonging to the class *Methanomicrobia*. In contrast, the first discovery of archaea in Antarctic cryoconite holes was mainly linked with *Thaumarchaeota* (Cameron et al., 2012). In benthic microbial mats of Lake Untersee, *Halobacteria* and *Methanobacteria* were dominant. Their presence was also reported from the moat region of Lake Fryxell (Brambilla et al., 2001) and *Halobacteria* were also detected in the hypersaline Antarctic Deep Lake in the Vestfold Hills (Bowman et al., 2000).

Archaeal RSVs in cryoconite holes accounted for 9.42% of all amplicon counts and were primarily affiliated with *Euryarchaeota*. Abundant RSVs belonging to *Methanomicrobia* were also present in lake sediments, suggesting that the glacier could be a biotic source for Lake Untersee not only for bacteria but also for archaea.

### Consequences of Climatic Changes in the Untersee Oasis

By the end of this century, ice-free regions in Antarctica could increase by 25% (Lee et al., 2017). Newly exposed rocks and soils will decrease the overall reflectance and hence absorb more solar energy resulting in higher melt rates supporting microbial life in cold and oligotrophic environments. Considering the climatic history that led to the present setting of Lake Untersee and the Anuchin Glacier (Schwab, 1998), the rate of increase in glacier retreat may be considered as a fast one (Figure 3). The average temperature during the austral summer fieldwork season 2015 already approximated 0°C. Further, daily temperature maxima spiked above the freezing point which was also evident in the long-term meteorological dataset (Andersen et al., 2015) and in 1996, a rain event was witnessed (Schwab, 1998).

Estimates show that rising temperatures will lead to substantial changes only within a few decades. As a consequence, moat regions [as observed in perennially ice-covered lakes of the McMurdo Dry Valleys, e.g., Lake Fryxell (Brambilla et al., 2001; Hall et al., 2017)] would enhance the rate of biotic input from supraglacial habitats into the lake, enable atmospheric exchanges and the pH of the waterbody would be reduced by the introduction of dissolved CO_2_ because of the formation of meltwater channels on the glacier (Andersen et al., 2015). These chemical and physical changes would most likely affect microbial communities of the Oasis. Further, satellite-based surface temperature data (Supplementary Figure 5) already indicate that the Anuchin Glacier and the ice cover of Lake Untersee are warmer compared to ice surfaces adjacent to the Oasis. This warming effect may be explained by katabatic winds as described in other areas of Antarctica (Lenaerts et al., 2016) and require further investigation in context with water availability for microbial life.

## Conclusion

The Anuchin Glacier harbors bacterial and archaeal communities in cryoconite holes and surface glacier ice. Based on the observed species and the Shannon index, we showed that both supraglacial habitats are significantly different from each other with respect to their microbial community. On average, cryoconite holes covered only 3.46% of the Anuchin glacier and hence we inferred that the volumetric fraction of cryoconite holes to the annual glacial melt volume was low. In this context, we consider the proportion of cryoconite biota as source for benthic microbial mats within Lake Untersee as substantial while the fraction of supraglacial ice was a neglectable biotic source for the microbiome of Lake Untersee. This finding is rather surprising considering the masses of ice that eventually drain into the lake and emphasizes the potential role of still unexplored englacial and subglacial zones.

Regarding this source tracking analysis, pandora’s box about which habitat is the source and which the sink is extremely tricky to open. As it has been shown in other studies that microbes traveling in the air are still viable and are hence biotic inoculators of other habitats microbial settlement is in a constant loop. It is not simply a downstream inoculation but might be recycled repeatedly. However, the main vector might still be deriving from cryoconite environments which may act as a determining factor for the microbiota in the lake.

Despite the presence of the thick ice cover of Lake Untersee which slows down shuttling of matter into the lake, cryoconite holes can be considered as biotic inoculation vectors that enable an indirect transfer of biota from soils and aerosols to the lake.

Here, cryoconite holes shared more genera with supraglacial ice (74.7%) than with aerosols and soils combined (53%) and therefore we reject our initial hypothesis that cryoconite hole microbial communities were dominated by microbial assemblages found in aerosols and adjacent soils. Further, from 150 genera in cryoconite holes, 69.3% of them were also present within Lake Untersee (including the lake ice cover). Considering this finding in combination with the source tracking analysis, we accept our hypothesis (b) that cryoconite hole microbial communities are mirrored in Lake Untersee environments.

The combination of wind driven temperature changes and global rising temperatures makes the Lake Untersee Oasis prone to rapid changes compared to other Antarctic regions. Rising temperatures will increase the availability of liquid water and hence induce glacio-morphological changes, reshaping habitats and will possibly alter the microbiome of the oasis. These changes require thorough monitoring to fully understand potential shifts in the microbiome. For this purpose, the present study has provided a reference database for future studies in context with climate change at Lake Untersee Oasis.

## Author Contributions

KW designed the study and collected samples (except benthic microbial mat samples that were collected by DA) during the Antarctic expedition 2015 that was led by DA. KW, and AP extracted DNA and did all subsequent genome analyses. KW and BS wrote most of the manuscript that was reviewed by all authors. Statistical analyses were done by AP.

## Conflict of Interest Statement

The authors declare that the research was conducted in the absence of any commercial or financial relationships that could be construed as a potential conflict of interest.
