# Supplementary material for: Source Environments of the Microbiome in Perennially Ice-Covered Lake Untersee, Antarctica
Source: Front Microbiol. 2019 May 10;10:1019. doi: 10.3389/fmicb.2019.01019 (PMC6524460; doi:10.3389/fmicb.2019.01019)
Supplement: Supplementary file 1 [file Data_Sheet_1.docx]

Supplementary Material

Source environments of the microbiome in perennially ice-covered Lake Untersee, Antarctica

Klemens Weisleitner*, Alexandra Perras, Dale T. Andersen, Christine Moissl-Eichinger, Birgit Sattler

*** Correspondence:** Birgit Sattler: [Birgit.Sattler@uibk.ac.at](mailto:Birgit.Sattler@uibk.ac.at)

**5 supplementary figures:**


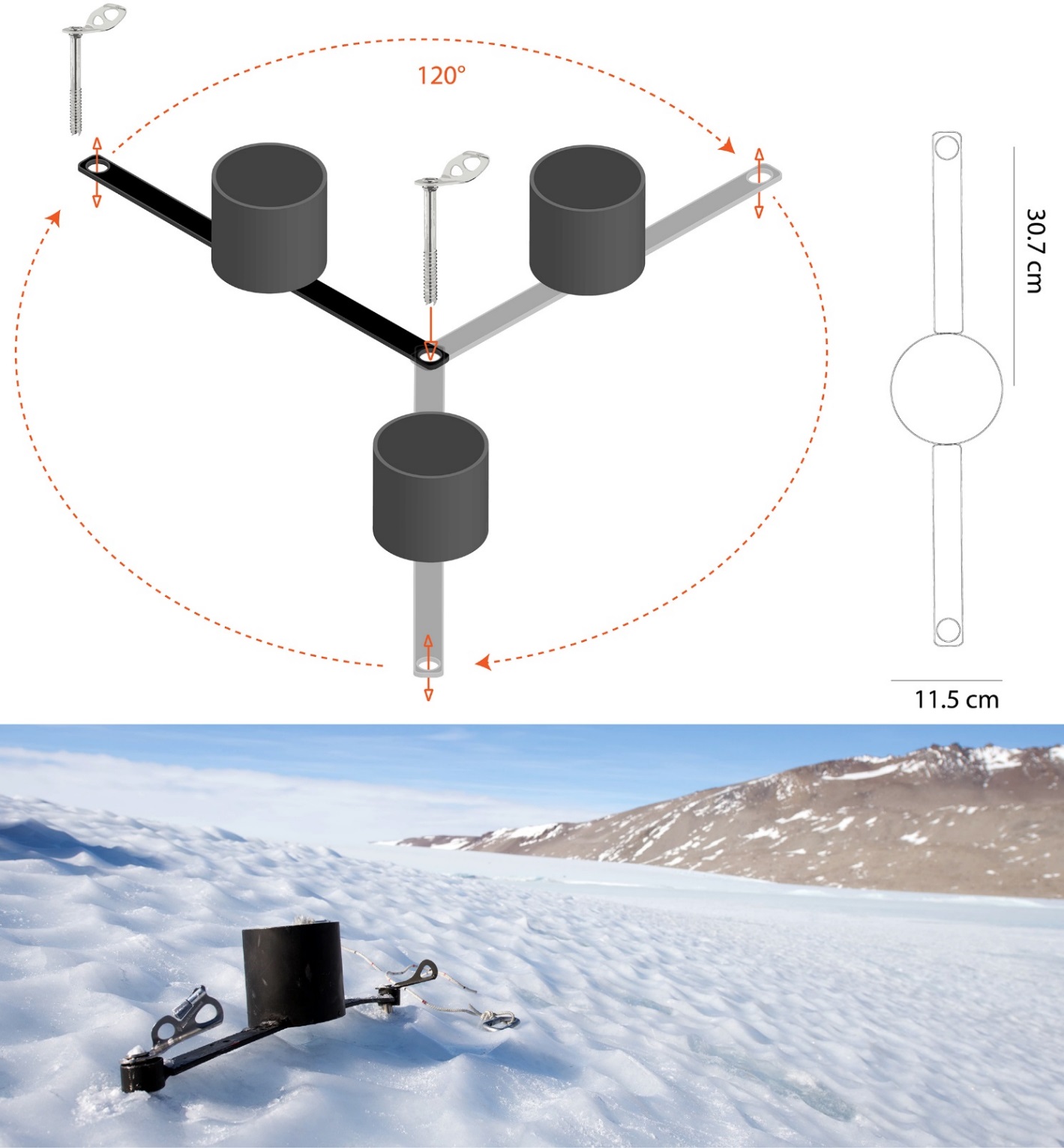


**Supplementary Figure 1:** TOP: Procedure of standardized ice core replicate retrieval and dimensions of the drill guidance system designed for Kovacs Mark III core barrels. The distance of the center of the ice cores to each other is 53.7 cm. BOTTOM: In-situ picture of the system. Even on rough slopes vertical coring instead of a perpendicular angle to the slope is possible. The guidance-system was also used to target for cryoconite holes.


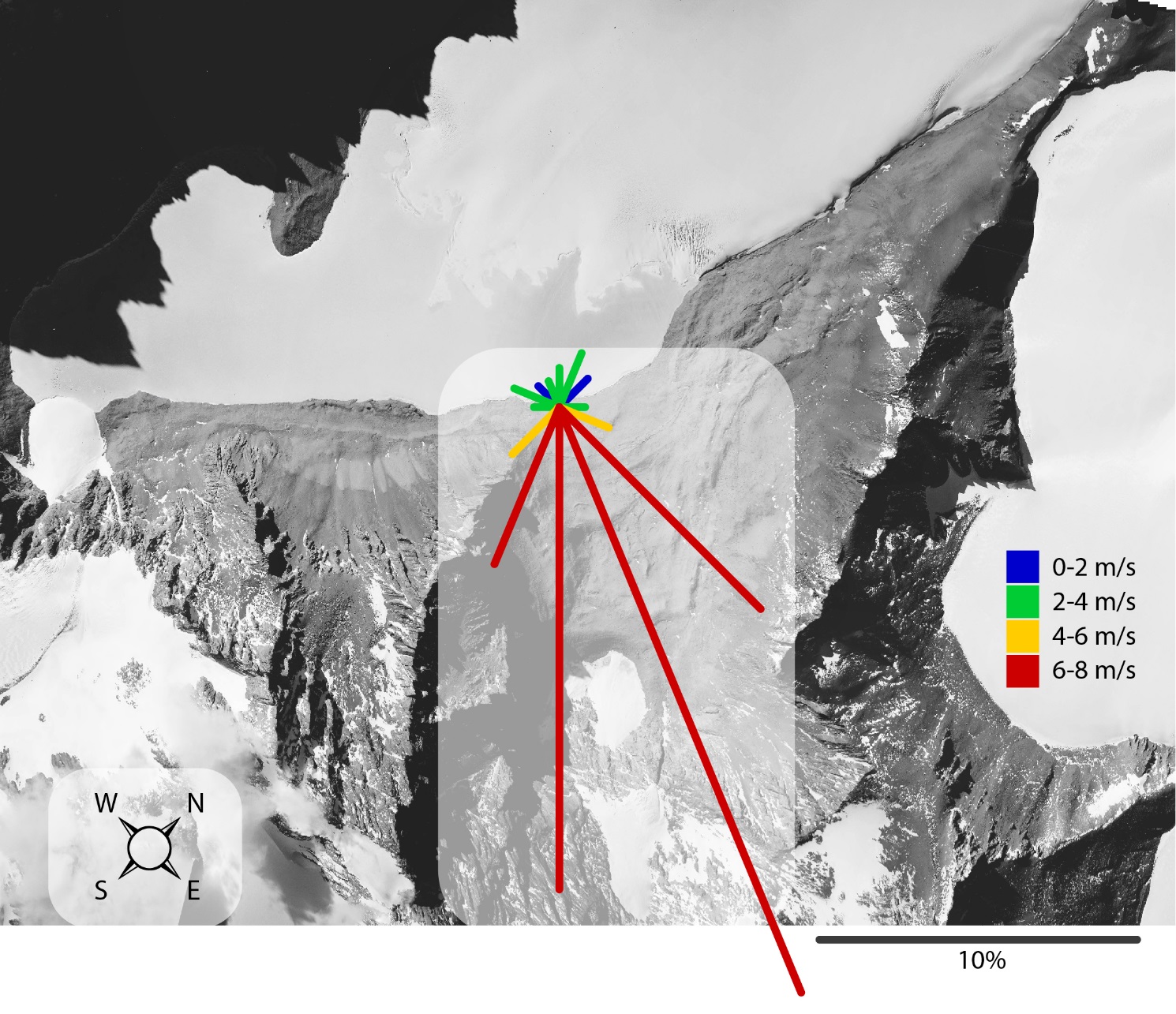


**Supplementary Figure 2:** Data from a custom-built weather station is plotted on an aerial image (© Bundesamt für Kartographie und Geodäsie, Frankfurt am Main, Germany). Each line indicates the wind direction and the corresponding average wind speed (color code). The relative duration of each wind direction is displayed as the magnitude of the vectors.


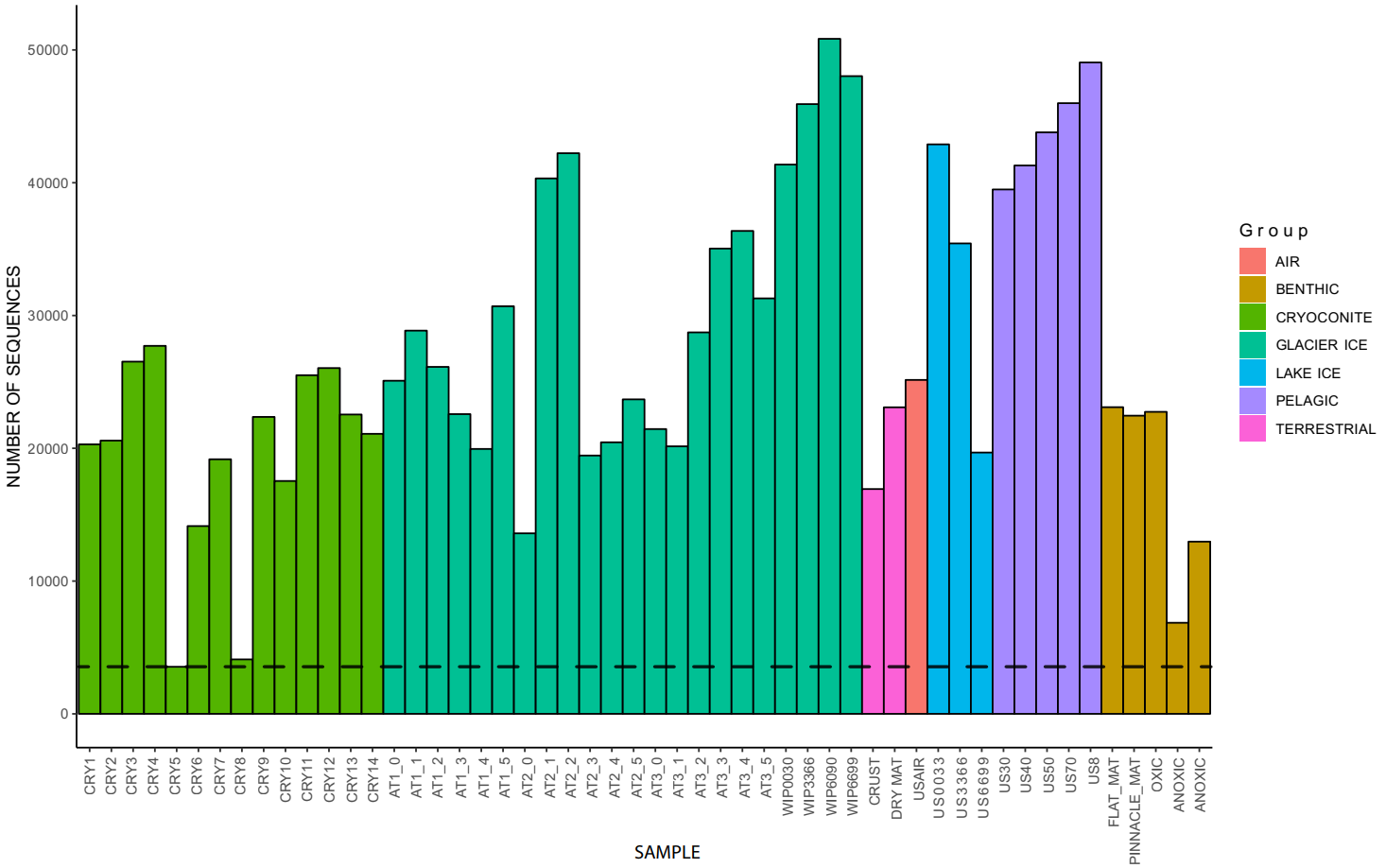


Supplementary Figure 3: Number of amplicon read counts per sample. The dashed line indicates the lowest number of sequences from all samples (3545 counts, CRY5).


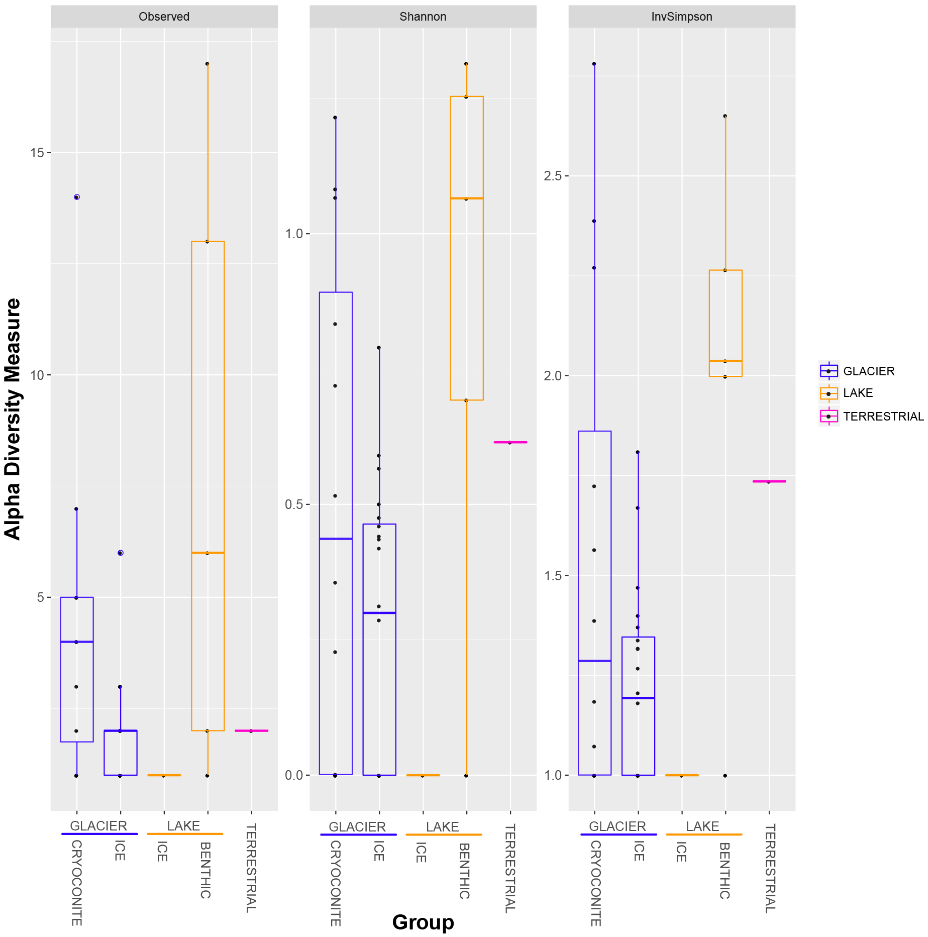


**Supplementary Figure 4:** The archaeal alpha diversity is shown for the indices “Observed”, “Shannon” and “InvSimpson”.

**
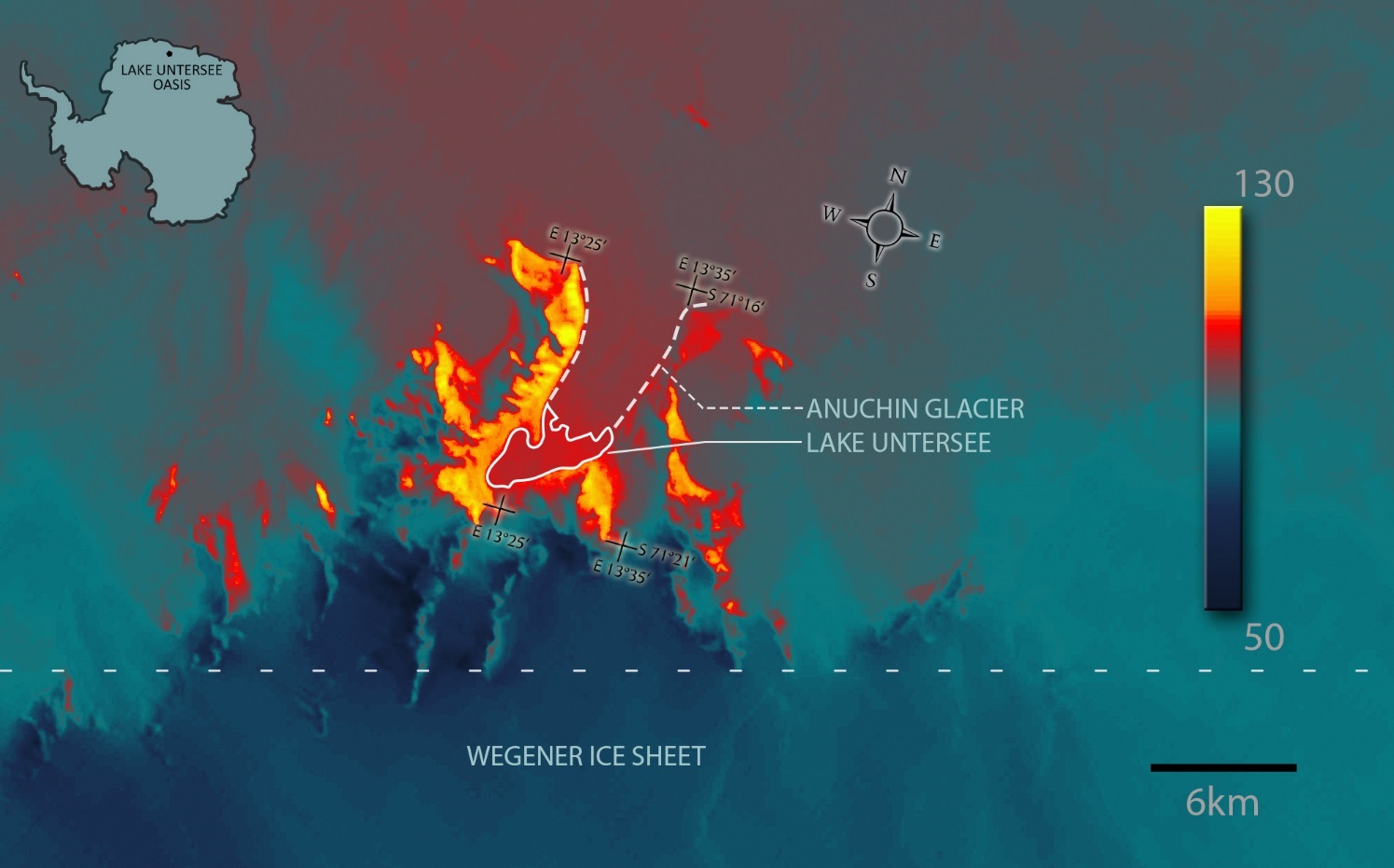
**

Supplementary Figure 5: Landsat 8 scene showing relative surface temperatures, captured on 20.10.2017. The black and white infrared channel was color mapped in a range of 50 to 130 units. Sun-exposed mountains appear as warmest and the Wegener Ice Sheet south of the oasis (below the horizontal dashed line) as coldest areas while the lake ice cover appears as warmest ice, respectively.
